# Effects of TheraBand and Theratube Eccentric Exercises on Quadriceps Muscle Strength and Muscle Mass in Young Adults

**DOI:** 10.1155/2021/5560144

**Published:** 2021-05-25

**Authors:** Shahnawaz Anwer, Shayista I. Jeelani, Sohrab Ahmad Khan, Nishat Quddus, Sumit Kalra, Ahmad H. Alghadir

**Affiliations:** ^1^Rehabilitation Research Chair, College of Applied Medical Sciences, King Saud University, Riyadh, Saudi Arabia; ^2^Department of Building and Real Estate, The Hong Kong Polytechnic University, Kowloon, Hong Kong SAR, China; ^3^Department of Rehabilitation Sciences, School of Nursing Sciences & Allied Health, Jamia Hamdard, New Delhi, India; ^4^Department of Physiotherapy and Rehabilitation Sciences, Max Super Specialty Hospital, New Delhi, India

## Abstract

**Purpose:**

This study was aimed at comparing the effects of TheraBand and theratube eccentric exercises on quadriceps muscle strength in young adults.

**Methods:**

Thirty young adults (19 females, 11 males) participated in this pretest-posttest experimental study. Participants were randomly assigned to one of the two groups: TheraBand and theratube groups. They received the training intervention 3 times a week for 4 weeks (12 sessions) with progression after 2 weeks. Maximum eccentric quadriceps strength was assessed using the Biodex isokinetic dynamometer system. Additionally, quadriceps muscle mass was measured using a tape.

**Results:**

Both groups showed a significant improvement in the peak torque of the eccentric isokinetic quadriceps' strength after weeks 2 and 4. Strength change in the quadriceps was nonsignificant in the theratube group compared to the TheraBand group after 4 weeks of training (*p* < 0.05). There was no increase in muscle mass during the 4 weeks of training in any group (*p* > 0.05).

**Conclusion:**

Both the TheraBand and theratube are equally effective in the strengthening of the quadriceps muscle in young adults. Therefore, either the TheraBand or theratube may be used according to the availability and feasibility of the subjects for training intervention.

## 1. Introduction

Stability of the knee joint during locomotion is mainly supported by the lower limb muscles. The quadriceps muscle may easily get fatigued due to weakness, resulting in poor neuromuscular control causing pathological movement at the knee joint. Additionally, weakness may also decrease the shock absorption capacity, resulting in increased loading on the knee joint. Consequently, quadriceps dysfunction may contribute to early degenerative changes [1].

Results of past studies have suggested that quadriceps weakness is a modifiable risk factor for progressive and symptomatic knee osteoarthritis (OA) [1–3]. Previous studies have suggested reduced quadriceps muscle mass in people with knee OA compared to age-matched controls [4, 5]. A growing body of evidence reported a positive correlation between muscle strength and mass [1–3]. While weakness in the quadriceps muscle can be functionally significant, it may be a potential risk factor for causing early onset knee OA [6, 7]. Some studies reported weakness of the quadriceps muscle prior to the development of knee OA. For instance, Omori et al. [8] reported weakness of quadriceps strength in asymptomatic women with radiographic knee OA with no pain or muscle atrophy. Additionally, a previous systematic review indicates quadriceps muscle weakness, which is a general risk factor causing the development of knee OA [9].

Resistance or strength training of the quadriceps has been used to reduce pain and improve knee function in people with knee OA [10]. There is a correlation between knee pain and muscle weakness as knee pain may cause atrophy and weakness of surrounding muscles [11]. It is also suggested that a reduced quadriceps sensory and motor function may result in the progression of knee OA [12]. Another study found weakness of quadriceps muscles in the absence of knee pain or muscle atrophy, which indicates that the quadriceps weakness may be due to muscle dysfunction [13]. Therefore, it was suggested that the weakness of the quadriceps muscle was possibly a primary risk factor for increased knee pain, reduced function, and progressive joint damage in people with knee OA [14].

While there is no direct evidence to support the positive effects of quadriceps strength training on the incident or progression of knee OA, strength training may reduce pain and improve knee function in people with knee OA [15]. Therefore, more interventional studies are required to examine the role of strength training regimens in reducing the risk of knee OA and protecting against functional loss in people with knee OA [1]. Hostler et al. [16] evaluated the effects of a resistance training program using an elastic band in young adults, and they found skeletal muscle adaptations following a short-term resistance training. However, weak muscles may find difficulty to perform resistance training using an elastic band, especially near the end of the range of motion, where the muscle is in a shortened position and the resistance of the material is greater [17]. The low cost and easy availability of elastic bands or tubes make this a highly versatile tool for resistance training during a therapeutic exercise program. Additionally, they are also useful for home-based therapeutic exercise convenient for people with knee OA [18, 19]. Furthermore, Mikesky et al. [20] suggest that home-based resistance training using elastic tubing is feasible and effective to improve muscle strength for older adults (average age = 71.2 years). Patterson et al. [21] quantified elastic resistance of TheraBand tubing during knee rehabilitation exercise. A recent systematic review suggests that elastic resistance training can promote strength gains comparable to conventional resistance training [22]. More recently, a study was conducted in Malaysia, which showed that variable resistance training with resistive bands produced significantly better strength and endurance compared to combined weight and chain exercise in healthy adults [23]. The TheraBand® product's affordability, availability, and portability make it ideal for worksite training, rehabilitation in hospitals, or in-home use. Therefore, primarily, this study examined the effects of TheraBand and theratube eccentric exercises on quadriceps muscle strength in healthy young adults. Secondarily, this study evaluated the effects of TheraBand and theratube eccentric exercises on quadriceps muscle mass in healthy young adults.

## 2. Materials and Methods

### 2.1. Participants

Thirty asymptomatic healthy collegiate athletes (19 females, 11 males; age 18 to 30 years) from the department of rehabilitation sciences, Hamdard University, New Delhi, India, participated in this pretest-posttest experimental study. Each participant was screened by a physician to rule out any medical conditions that may preclude to participate in the exercise intervention. Individuals with a history of trauma to the lower extremities, systemic inflammation (e.g., rheumatoid arthritis and gout), or low back pain or who participated in a knee rehabilitation program in the last 6 weeks were excluded. The study protocol was approved by the research ethics committee, Hamdard University, New Delhi. A written informed consent was obtained from each participant.

### 2.2. Study Design

**Figure 1:**
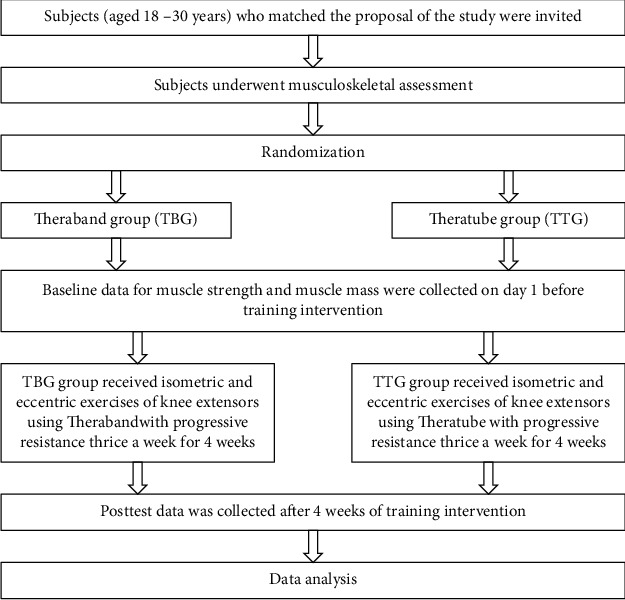
Study procedure.

The study was a two-group pretest and posttest experimental design. Participants were randomly assigned to one of the two groups: TheraBand (TBG, *n* = 15) and theratube (TTG, *n* = 15) using a computer-generated random number [24]. Both groups received eccentric exercise of the knee for 4 weeks (Figure 1).

### 2.3. Outcome

#### 2.3.1. Measurement of Muscle Mass

The muscle's circumference of the thigh was measured by a measuring tape and compared to determine the amount of quadriceps muscle mass in the dominant leg. Measurement of thigh circumference was performed at 15 cm proximal to the superior pole of the patella [25]. Measurements were taken at baseline, week 2, and week 4.

#### 2.3.2. Measurement of Eccentric Strength of the Quadriceps Muscle

**Figure 2:**
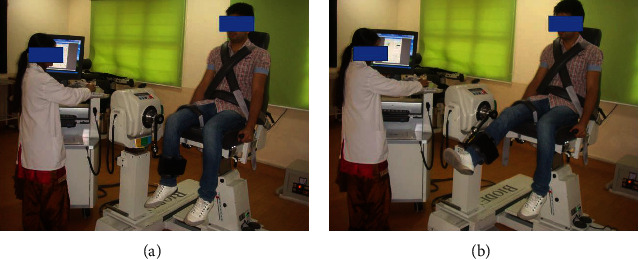
Measurement of quadriceps peak torque using Biodex isokinetic dynamometer System 4: (a) starting and (b) end positions.

Eccentric strength of the quadriceps muscle (e.g., peak torque) in the dominant leg was measured using a calibrated Biodex isokinetic dynamometer (System 4) (Figure 2). The subjects were made to sit in a position such that the backrest reclined 5° from the vertical and the knees flexed at 90°. The straps were fastened to stabilize the subject's thigh, pelvis, and trunk to avoid compensatory movements. The dynamometer axis and lever arm were aligned with the axis of knee rotation (e.g., lateral femoral epicondyle) and distal leg (5 cm above the medial maleolus), respectively, to allow full ankle dorsiflexion [26]. The eccentric peak torque of the knee extensors of the dominant leg was recorded at 60°/s. Three maximal attempts were made, and the highest value was used for data analysis. Measurements were taken at baseline, week 2, and week 4.

### 2.4. Description of TheraBand and Theratubes

Elastic resistive bands such as TheraBands and theratubes are commonly used to improve muscle strength in rehabilitation. Both the band and tube display similar resistance properties, for example, the resistance increases as the band or tube is stretched. In essence, the force produced by the bands or tubes depend on three factors including the elastic coefficient, cross-sectional area, and change in length as given in the following formula: *F* = *k*∗CSA∗Δ*L*, where *k* is a constant, CSA is the increase in cross-sectional area, and Δ*L* is the change in length [27]. The percentage change in resting length can be calculated using the formula as follows:

(1) $$\begin{array}{c} \%\Delta L=\left[\frac{\text{Final length}-\text{Resting length}}{\text{Resting length}}\right]*100. \end{array}$$

**Figure 3:**
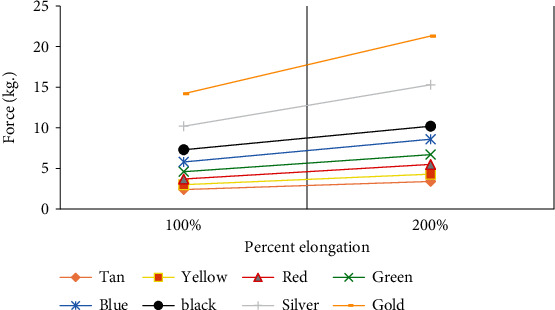
Force production of TheraBand or theratubes after 100 and 200% elongation.

In general, TheraBand or theratubes are available in 8 different colors (e.g., tan and yellow colors for beginners which produce the least resistance and silver and gold colors for advanced users which produce maximum resistance) as per their elastic resistance. For instance, a 1-meter length of yellow band or tube stretched to 100% (i.e., double the resting length) would produce about 1.36 kg of elastic resistance (Table 1) [28]. Each progressive band or tube produces 25% and 40% higher pull force in the clinical range (Figure 3) (tan through black) and the advanced range (silver through gold), respectively [28].

### 2.5. Interventions

#### 2.5.1. TheraBand Group (TBG)

Participants in this group used TheraBand exercise bands and performed exercises in standard positions and were progressed through the color hierarchy (Table 1). TheraBand has different color coding according to the level of resistance. Participants begun with the less resistive bands (e.g., yellow band) to perform two sets of 10 repetitions of each exercise in the correct manner. Consequently, they progressed to the higher resistance exercise band (e.g., red band) and performed each exercise 3 sets of 10 repetitions. As the exercises with resistive bands at one level became easier to perform, participants were asked to progress to the next level of exercise (exercise progression: yellow band in the first two weeks and red band in the last two weeks). Participants in this group received a 4-week, thrice-weekly, individualized, supervised exercise program as described below [29, 30].

**Figure 4:**
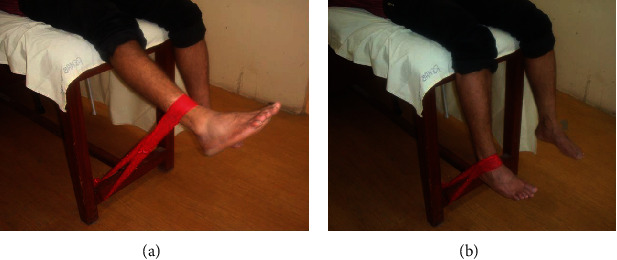
Eccentric knee extensor exercise with TheraBand or theratube: (a) starting and (b) end positions.

- *Static quadriceps exercise*: the participant was in a supine position, and he was asked to contract the quadriceps muscle and push the knee downwards while maintaining the foot in full dorsiflexion
- *Straight leg raise exercise*: the participant was made to lie on his back on the treatment couch. He was asked to bend his dominant knee to a 90-degree angle, and with the foot flat on the surface, the dominant leg was lifted slowly away from the couch up to 20 degrees and held it for 10 seconds
- *Quadriceps extensor exercise*: participant was asked to sit on the quadriceps chair and extend his dominant leg straight where the quadriceps muscle is pulled while maintaining the foot in full dorsiflexion and hold it for 10 seconds
- *Eccentric knee extension exercise*: participant was made to lie in a supine position, and his dominant knee was at the edge of the plinth (Figure 4). While lying, the elastic TheraBand was placed underneath the foot. The participant held on to the ends and straightened the knee to full extension. The participant was asked to flex the knee towards the resistance as slowly as possible so that the quadriceps was contracting eccentrically

#### 2.5.2. Theratube Group (TTG)

Participants in this group used the theratube and performed exercises in standard positions and were progressed through the color hierarchy. Various resistance levels of the tubes were provided, which were colors coded according to their resistance level. Exercises using theratubes were initially performed for 2 sets of 10 repetitions using the lightest resistance (yellow) [29]. When the participant was able to perform 3 sets of 10 repetitions without fatigue, they progressed to increased resistance (red) and worked up with 3 sets of 10 repetitions [29]. Participants in TTG were asked to perform the same set of exercises (e.g., isometric and eccentric knee exercises) using the theratube for resistance as described above for the TBG [29, 30].

## 3. Data Analysis

Data was analyzed using the SPSS (version 16.0, SPSS Inc., Chicago, IL). Independent *t*-test was used to compare the changes in eccentric quadriceps strength and muscle mass between the groups. Repeated measure analysis of variance (ANOVA) was used for within-group analysis to study the changes in eccentric quadriceps strength and muscle girth. A statistically significant difference was defined as a *p* value less than 0.05.

## 4. Results

### 4.1. Demographic Data

A total of 30 subjects (19 females, 11 males) participated in the study and completed the training interventions. Their demographic data was analyzed by comparing means of descriptive analysis. Their age, height, and weight were recorded. The mean age (standard deviation, SD) of TBG and TTG was 24.6 (1.34) and 24.7 (1.22) years, respectively. The mean height (SD) of TBG and TTG was 162.4 (9.76) and 157.9 (8.09) cm, respectively. The mean weight (SD) of TBG and TTG was 59.8 (9.90) and 59.7 (14.52) kg, respectively.  Table 2 gives details of the descriptive statistics of the demographic data. These variables had insignificant differences between the two groups.

### 4.2. Eccentric Isokinetic Quadriceps Strength

The data analysis revealed a nonsignificant difference in peak torque of the eccentric isokinetic quadriceps' strength at baseline, week 2, and week 4 between the two groups (*p* = 0.075, 0.421, and 0.322, respectively). Within-group comparison showed a significant improvement in the peak torque of the eccentric isokinetic quadriceps' strength after week 2 in each group. The improvements in peak torque of the eccentric isokinetic quadriceps' strength remained significant after week 4 in each group (Table 3).

### 4.3. Muscles Mass

The data analysis revealed nonsignificant differences in the quadriceps muscle mass at baseline, week 2, and week 4 between the two groups (*p* = 0.705, 0.437, and 0.432, respectively). Similarly, within-group analysis showed insignificant changes in the quadriceps muscle mass over 4 weeks of training (Table 4).

## 5. Discussion

The present study was conducted to compare the effects of TheraBand and theratube eccentric exercises in the improvement of quadriceps muscle strength in young adults. This study proposed to integrate strength training with exercise and elastic resistance to see its effect on quadriceps strength. It was hypothesized that the use of TheraBand or theratube for eccentric exercises would be an effective strategy to improve the strength of the quadriceps muscle in young adults. The results of the current study indicate that both TheraBand and theratube are equally effective in the improvement of quadriceps muscle strength over 4 weeks of training.

Many studies showed that most of the evidence indicated that elastic resistance training can be an effective tool to increase the strength of the quadriceps muscles [31–33]. Elastic resistance training either by TheraBand or by theratube with voluntary exercise has shown significant improvement in strength in healthy subjects with and without musculoskeletal pain [34, 35].

The possible mechanism for strength gain during this training can be explained by the neural adaptation mechanism [36]. If there is an increase in muscular strength with no obvious hypertrophy, it indicates the involvement of neural adaptation for gaining muscular strength. A previous study examined strength gain using surface electromyography (sEMG), and they found a significant association between strength gain in the early phase of training and the increase in the amplitude of sEMG activity [37]. This type of strength gain is primarily due to an increase in neural drive, which represents the magnitude of the efferent neural output of active muscle fibers. While sEMG activity denotes the amount of muscle activity, changes in sEMG activity are the result of various motor unit firing patterns as measured by electrodes. A few studies have found a transient increase in the motor unit firing rate [38, 39]. Training-dependent increases in the rate of tension development have also been associated with an increased firing rate in individual motor units. Motor unit synchronization is another proposed mechanism causing increased muscle strength; however, it is yet to be examined [40].

There are the following suggestions recommended considering the existing evidence. First, while mental practice (e.g., imagined contractions) may help increase the excitability of the cortical areas that are used for movement and motion planning [41], the effects of such training are not better with physical training. Therefore, it is suggested to incorporate motor learning theory and imagined contractions into strength training practice for an effective exercise regime [42]. Second, since the length of the muscle is important for muscle contraction, static contraction should be practiced at greater muscle lengths to increase force production [43]. Third, in the presence of muscle pain, immobilization, or detraining, submaximal eccentric contraction can be used [44]. Fourth, in the case of older adults, especially to reduce fall risk, the reversal of the antagonist (antagonist-to-agonist) proprioceptive neuromuscular facilitation contraction pattern may be used to improve the rate of tension development in the muscles [37]. Fifth, in sEMG recordings, it is recommended to measure antagonist EMG activity to evaluate the neural changes induced by strength training [45].

With regard to the secondary aim of this study, both TheraBand and theratube eccentric exercises yield no change in muscle mass after the 4 weeks of training in any group. The factors that reveal the mechanism state of the strength gain in response to strength training are primarily determined by two aspects, the morphological characteristics of the muscles and the neural activation of the muscles. The neural activation of the muscles can be altered by the number of motor units that are recruited, the firing frequency of each motor unit, and the coordination between synergistic muscles and inhibitory reflexes [36, 46]. Those participants who have no previous experience in training usually demonstrate a rapid increase in strength during the first few weeks [47]. This rapid change in strength is commonly attributed to neural adaptations, because often no significant alteration in muscle size can be found during this period [36]. Muscle hypertrophy may become measurable only after several weeks of training [47], although it has been reported that the concentration of contractile proteins may be upregulated even after a single training session [48].

### 5.1. Clinical Implications

Muscle weakness plays a major role in the pathogenesis of many diseases of the knee such as osteoarthritis (OA), even in the early stages of the disease; therefore, emphasis should be laid on the strengthening of the quadriceps muscles. Elastic resistance training could be an effective intervention to improve muscle strength, especially during a home exercise program when individuals such as an elderly who may not be able to attend the rehabilitation center for strength training [49, 50]. Since the current study demonstrated a significant change in the strength of the quadriceps femoris muscle after 4 weeks of strength training with elastic resistance, it may reduce the treatment costs and rehabilitation time for patients as well as the therapist.

### 5.2. Study Limitations

This study had some potential limitations. This study used only two colors of TheraBand and theratube. As different colors of these elastic bands produced different resistance, so the color code of higher resistance might produce a significant difference between the two bands. Elastic resistance training is an area that needs to be explored more with special attention given to strengthening in healthy individuals. Although many studies have investigated the effects of TheraBand on muscle strength, few studies evaluated the effects of theratube on muscle strength. Additionally, no studies have compared the effectiveness of these two resistive techniques on muscle strength. Since the present work examined the effects of 4 weeks of training, it can be said that the effects were mainly due to neuromuscular adaptations. Future studies are warranted to examine the long-term effects of TheraBand or theratube resistive training programs on muscle hypertrophy. Additionally, the current study only measured quadriceps muscle strength after TheraBand and theratube resistance training. Future studies may examine other muscle groups such as hamstring and upper limb muscles (e.g., biceps and triceps).

## 6. Conclusions

This study concludes that both the TheraBand and theratube can be used for the strengthening of the quadriceps muscle according to the availability and feasibility of the training intervention. The study also indicates no increase in muscle mass of the quadriceps during the 4 weeks of training intervention using either the TheraBand or the theratube. More randomized controlled trials are needed to confirm these results.

## Figures and Tables

**Table 1:** Progression of TheraBand or theratubes.

TheraBand® band or tube color	Increase from preceding color at 100% elongation	Thickness	Resistance at 100% elongation (kg)
Tan	—	Very thin	1.09
Yellow	25%	Thin	1.36
Red	25%	Medium	1.68
Green	25%	Heavy	2.09
Blue	25%	Extra heavy	2.63
Black	25%	Special heavy	3.31
Silver	40%	Super heavy	4.63
Gold	40%	Advanced heavy	6.44

**Table 2:** Demographic data.

	TBG mean ± SD	TTG mean ± SD	Descriptive analysis
No. of subjects	15	15	*p* value
Age (years)	24.6 ± 1.34	24.7 ± 1.22	0.888
Height (cm)	162.4 ± 9.76	157.9 ± 8.09	0.177
Weight (kg)	59.8 ± 9.90	59.7 ± 14.52	0.988

TBG: TheraBand group; TTG: theratube group.

**Table 3:** Between and within-group comparisons of eccentric isokinetic quadriceps' strength.

	TBG mean ± SD	TTG mean ± SD	*p* value (*t*-test)
Baseline	75.4 ± 10.03	67.6 ± 12.99	0.075
Week 2	78.6 ± 8.46^∗^	76.5 ± 5.82^∗^	0.421
Week 4	88.9 ± 17.92^∗∗^	83.8 ± 8.43^∗∗^	0.322
*p* value (ANOVA)	At week 2, 0.014^∗^; at week 4, 0.005^∗∗^	At week 2, 0.002^∗^; at week 4, 0.001^∗∗^	

TBG: TheraBand group; TTG: theratube group.

**Table 4:** Between and within-group comparisons of muscle mass at dominant thigh.

	TBG mean ± SD	TTG mean ± SD	*p* value (*t*-test)
Baseline	46.2 ± 3.34	47.9 ± 7.83	0.705
Week 2	46.2 ± 3.34	47.9 ± 7.83	0.437
Week 4	46.2 ± 3.34	47.9 ± 7.83	0.432
*p* value (ANOVA)	>0.05	>0.05	

TBG: TheraBand group; TTG: theratube group.

## Data Availability

The data set of this manuscript is available on reasonable request through the corresponding author.

## References

[1] Segal N. A., Glass N. A. (2011). Is quadriceps muscle weakness a risk factor for incident or progressive knee osteoarthritis?. *The Physician and sportsmedicine*.

[2] Lewek M. D., Rudolph K. S., Snyder-Mackler L. (2004). Quadriceps femoris muscle weakness and activation failure in patients with symptomatic knee osteoarthritis. *Journal of Orthopaedic Research*.

[3] Glass N. A., Torner J. C., Law L. F. (2013). The relationship between quadriceps muscle weakness and worsening of knee pain in the MOST cohort: a 5-year longitudinal study. *Osteoarthritis and cartilage*.

[4] Ikeda S., Tsumura H., Torisu T. (2005). Age-related quadriceps-dominant muscle atrophy and incident radiographic knee osteoarthritis. *Journal of Orthopaedic Science*.

[5] Segal N. A., Toda Y. (2005). Absolute reduction in lower limb lean body mass in Japanese women with knee osteoarthritis. *Journal of Clinical Rheumatology*.

[6] Roos E. M., Herzog W., Block J. A., Bennell K. L. (2011). Muscle weakness, afferent sensory dysfunction and exercise in knee osteoarthritis. *Nature Reviews Rheumatology*.

[7] Bennell K. L., Hunt M. A., Wrigley T. V., Lim B. W., Hinman R. S. (2008). Role of muscle in the genesis and management of knee osteoarthritis. *Rheumatic Disease Clinics of North America*.

[8] Omori G., Narumi K., Nishino K. (2016). Association of mechanical factors with medial knee osteoarthritis: a cross-sectional study from Matsudai Knee Osteoarthritis Survey. *Journal of Orthopaedic Science*.

[9] Øiestad B. E., Juhl C. B., Eitzen I., Thorlund J. B. (2015). Knee extensor muscle weakness is a risk factor for development of knee osteoarthritis. A systematic review and meta-analysis. *Osteoarthritis and Cartilage*.

[10] Kristensen J., Franklyn-Miller A. (2012). Resistance training in musculoskeletal rehabilitation: a systematic review. *British journal of sports medicine*.

[11] Sattler M., Dannhauer T., Hudelmaier M. (2012). Side differences of thigh muscle cross-sectional areas and maximal isometric muscle force in bilateral knees with the same radiographic disease stage, but unilateral frequent pain - data from the osteoarthritis initiative. *Osteoarthritis and cartilage*.

[12] Topp R., Woolley S., Hornyak J., Khuder S., Kahaleh B. (2002). The effect of dynamic versus isometric resistance training on pain and functioning among adults with osteoarthritis of the knee. *Archives of Physical Medicine and Rehabilitation*.

[13] Slemenda C., Brandt K. D., Heilman D. K. (1997). Quadriceps weakness and osteoarthritis of the knee. *Annals of internal medicine*.

[14] Segal N. A., Glass N. A., Torner J. (2010). Quadriceps weakness predicts risk for knee joint space narrowing in women in the MOST cohort. *Osteoarthritis and Cartilage*.

[15] Vincent K. R., Vincent H. K. (2012). Resistance exercise for knee osteoarthritis. *PM&R*.

[16] Hostler D., Schwirian C. I., Campos G. (2001). Skeletal muscle adaptations in elastic resistance-trained young men and women. *European journal of applied physiology*.

[17] Santos G. M., Tavares G. M. S., de Gasperi G., Bau G. R. (2009). Mechanical evaluation of the resistance of elastic bands. *Brazilian Journal of Physical Therapy*.

[18] Chang T. F., Liou T. H., Chen C. H., Huang Y. C., Chang K. H. (2012). Effects of elastic-band exercise on lower-extremity function among female patients with osteoarthritis of the knee. *Disability and rehabilitation*.

[19] Cho I., Hwangbo G., Lee D., Lee S. (2014). The effects of closed kinetic chain exercises and open kinetic chain exercises using elastic bands on electromyographic activity in degenerative gonarthritis. *Journal of physical therapy science*.

[20] Mikesky A. E., Topp R., Wigglesworth J. K., Harsha D. M., Edwards J. E. (1994). Efficacy of a home-based training program for older adults using elastic tubing. *European journal of applied physiology and occupational physiology*.

[21] Patterson R. M., Caroline W., Jansen S., Hogan H. A., Nassif M. D. (2001). Material properties of Thera-Band tubing. *Journal of the American Physical Therapy Association*.

[22] Lopes J. S., Machado A. F., Micheletti J. K., De Almeida A. C., Cavina A. P., Pastre C. M. (2019). Effects of training with elastic resistance versus conventional resistance on muscular strength: a systematic review and meta-analysis. *SAGE open medicine*.

[23] Amir Bahram K., Kim G. S., Kim L. S., Swee L. O., Kittichottipanich B. (2020). Effects of 12 weeks combined weight and chain versus combined weight and elastic band variable resistance training on upper and lower body muscular strength and endurance among untrained males in Iran. *Malaysian Journal of Movement, Health & Exercise*.

[24] Alghadir A. H., Anwer S., Sarkar B., Paul A. K., Anwar D. (2019). Effect of 6-week retro or forward walking program on pain, functional disability, quadriceps muscle strength, and performance in individuals with knee osteoarthritis: a randomized controlled trial (retro-walking trial). *BMC musculoskeletal disorders*.

[25] Soderberg G. L., Ballantyne B. T., Kestel L. L. (1996). Reliability of lower extremity girth measurements after anterior cruciate ligament reconstruction. *Physiotherapy Research International*.

[26] Sole G., Hamrén J., Milosavljevic S., Nicholson H., Sullivan S. J. (2007). Test-retest reliability of isokinetic knee extension and flexion. *Archives of physical medicine and rehabilitation*.

[27] Rovny D. (2004). The scientific and clinical application of elastic resistance. *Physical Therapy*.

[28] Uchida M. C., Nishida M. M., Sampaio R. A., Moritani T., Arai H. (2016). Thera-band® elastic band tension: reference values for physical activity. *Journal of Physical Therapy Science*.

[29] Rabita G., Pérot C., Lensel-Corbeil G. (2000). Differential effect of knee extension isometric training on the different muscles of the quadriceps femoris in humans. *European journal of applied physiology*.

[30] Mulvany R., Zucker-Levin A. R., Jeng M. (2010). Effects of a 6-week, individualized, supervised exercise program for people with bleeding disorders and hemophilic arthritis. *Physical therapy*.

[31] Iversen V. M., Mork P. J., Vasseljen O., Bergquist R., Fimland M. S. (2017). Multiple-joint exercises using elastic resistance bands vs. conventional resistance-training equipment: a cross-over study. *European Journal of Sport Science*.

[32] Jakobsen M. D., Sundstrup E., Andersen C. H. (2012). Muscle activity during knee-extension strengthening exercise performed with elastic tubing and isotonic resistance. *International Journal of Sports Physical Therapy*.

[33] Janusevicius D., Snieckus A., Skurvydas A. (2017). Effects of high velocity elastic band versus heavy resistance training on hamstring strength, activation, and sprint running performance. *Journal of Sports Science & Medicine*.

[34] Jakobsen M. D., Sundstrup E., Andersen C. H., Aagaard P., Andersen L. L. (2013). Muscle activity during leg strengthening exercise using free weights and elastic resistance: effects of ballistic vs controlled contractions. *Human movement science*.

[35] Sundstrup E., Jakobsen M. D., Andersen C. H. (2014). Evaluation of elastic bands for lower extremity resistance training in adults with and without musculo-skeletal pain. *Scandinavian journal of medicine & science in sports*.

[36] Sale D. G. (1988). Neural adaptation to resistance training. *Medicine and science in sports and exercise*.

[37] Gabriel D. A., Kamen G., Frost G. (2006). Neural adaptations to resistive exercise. *Sports Medicine*.

[38] Pasquet B., Carpentier A., Duchateau J. (2006). Specific modulation of motor unit discharge for a similar change in fascicle length during shortening and lengthening contractions in humans. *The Journal of physiology*.

[39] de Luca C. J., Foley P. J., Erim Z. E. (1996). Motor unit control properties in constant-force isometric contractions. *Journal of Neurophysiology*.

[40] Enoka R. M., Christou E. A., Hunter S. K. (2003). Mechanisms that contribute to differences in motor performance between young and old adults. *Journal of Electromyography and Kinesiology*.

[41] Yue G., Cole K. J. (1992). Strength increases from the motor program: comparison of training with maximal voluntary and imagined muscle contractions. *Journal of Neurophysiology*.

[42] Paravlic A. H., Slimani M., Tod D., Marusic U., Milanovic Z., Pisot R. (2018). Effects and dose–response relationships of motor imagery practice on strength development in healthy adult populations: a systematic review and meta-analysis. *Sports Medicine*.

[43] McHugh M. P., Tetro D. T. (2003). Changes in the relationship between joint angle and torque production associated with the repeated bout effect. *Journal of Sports Science*.

[44] Vila-Chã C., Falla D., Farina D. (2010). Motor unit behavior during submaximal contractions following six weeks of either endurance or strength training. *Journal of Applied Physiology*.

[45] Kellis E., Katis A. (2007). The relationship between isokinetic knee extension and flexion strength with soccer kick kinematics: an electromyographic evaluation. *Journal of Sports Medicine and Physical Fitness*.

[46] Moritani T. (1979). Neural factors versus hypertrophy in the time course of muscle strength gain. *American journal of physical medicine*.

[47] Staron R. S., Karapondo D. L., Kraemer W. J. (1994). Skeletal muscle adaptations during early phase of heavy-resistance training in men and women. *Journal of Applied Physiology*.

[48] Willoughby D. S., Nelson M. J. (2002). Myosin heavy-chain mRNA expression after a single session of heavy-resistance exercise. *Medicine and science in sports and exercise*.

[49] Stojanović M. D., Mikić M. J., Milošević Z., Vuković J., Jezdimirović T., Vučetić V. (2021). Effects of chair-based, low–load elastic band resistance training on functional fitness and metabolic biomarkers in older women. *Journal of Sports Science & Medicine*.

[50] Fragala M. S., Cadore E. L., Dorgo S. (2019). Resistance training for older adults: position statement from the national strength and conditioning association. *The Journal of Strength & Conditioning Research*.

